# Editorial: Advance in translational genomics based biomarkers for cancer management: discovery and application

**DOI:** 10.3389/fgene.2023.1342616

**Published:** 2023-12-12

**Authors:** Guodong Zhao, Qinyu Ge, Wenjun Lan, Bo Zheng, Yuting Liang

**Affiliations:** ^1^ Zhejiang University Kunshan Biotechnology Laboratory, Zhejiang University Kunshan Innovation Institute, Kunshan, Jiangsu, China; ^2^ State Key Laboratory of Bioelectronics, School of Biological Science and Medical Engineering, Southeast University, Nanjing, China; ^3^ Cold Spring Harbor Laboratory, Cold Spring Harbor, NY, United States; ^4^ The Affiliated Suzhou Hospital of Nanjing Medical University, Suzhou Municipal Hospital, Gusu School, Nanjing Medical University, Suzhou, Jiangsu, China; ^5^ Center for Clinical Laboratory, The First Affiliated Hospital of Soochow University, Suzhou, Jiangsu, China

**Keywords:** translational genomics, biomarkers, cancer management, discovery, challenges

This editorial summarizes the contributions to the Research Topic “*Advance in Translational Genomics Based Biomarkers for Cancer Management: Discovery and Application*,” established under the Cancer Genetics and Oncogenomics section and appearing under the *Frontiers in Genetics*, *Frontiers in Immunology* and *Frontiers in Oncology* journals.

Cancer management demands robust biomarkers for early diagnosis, measuring disease progression, guiding targeted treatments, and avoiding drug resistance. The integration of genomic biomarkers has become indispensable, with mounting evidence supporting their ability to enhance traditional pathology, thereby improving clinical decision-making and outcomes. This Research Topic in *Frontiers in Genetics* aimed to compile cutting-edge original research on the discovery and application of translational genomics-based biomarkers across various cancer types.

One notable study (Stubbe et al.) by validated promoter hypermethylation of *SFRP1* (*phSFRP1*) in circulating cell-free DNA as a prognostic biomarker for overall survival in stage I-II pancreatic ductal adenocarcinoma (PDAC). The findings, based on 211 patients, indicated that *phSFRP1* significantly correlated with shorter survival, independently of other prognostic factors. Interestingly, *phSFRP1* did not impact recurrence, suggesting its potential to identify patients with limited benefit from adjuvant chemotherapy post-resection, rather than indicating a more aggressive tumor biology. R0-resected patients with *phSFRP1* had survival comparable to unresected patients without *phSFRP1*, questioning the value of resection in this subset. Overall, these clinically relevant findings propose *phSFRP1* as a valuable prognostic and potentially predictive blood-based biomarker in early-stage PDAC. If confirmed through larger validation studies, it could augment clinical decision-making regarding surgery *versus* upfront systemic therapy, and guide more personalized approaches to improve outcomes. As such, *phSFRP1* merits incorporation into future clinical trials examining both prognostic stratification and predictive response to adjuvant treatment in resectable PDAC. The mechanistic role of *phSFRP1* silencing also warrants further investigation as an actionable target for novel epigenetic therapies.

In a second study Pei et al. identified several potential DNA methylation markers for noninvasive detection of esophageal cancer (EC) and evaluated their performance in plasma. Testing seven cancer-related genes revealed significant differential methylation between 48 EC cases and 101 controls, with the combination of *ZNF582* and *FAM19A4* methylation showing the best diagnostic accuracy, with 60.4% sensitivity and 83.2% specificity. This two-gene panel also showed comparable performance across age and gender subgroups. When combined with CEA levels, the sensitivity improved to 71.1% while retaining a reasonable specificity of 75.8%. In an independent validation set, the panel demonstrated 60.0% sensitivity and 90.0% specificity for EC detection, significantly distinguishable from gastric or colorectal cancers. Overall, this study identifies a plasma methylation marker panel with potential utility for early noninvasive EC early detection/screening. If validated in larger multicenter trials, it could provide a cost-effective alternative to endoscopy-based screening to reduce EC incidence and mortality. The simplicity of measuring DNA methylation from blood samples further supports its feasibility as a routine test.

Another comprehensive review by Xue et al. summarized the current status and challenges of utilizing DNA methylation markers for noninvasive early detection of gastric cancer (GC). It provides an overview of DNA methylation changes in GC, sample types and analytical methods used, individual methylation markers evaluated, marker panels developed, and DNA methylation-based pan-cancer tests applied for GC early detection/screening. The authors highlight that most GC methylation biomarker studies are still in the early discovery stages focused on Asian populations. Key limitations are the lack of GC-specific methylation markers, insufficient sensitivity for detecting early-stage cancers, and need for large prospective multicenter validation in diverse asymptomatic cohorts. Overall, DNA methylation shows promise as a biomarker for GC screening but requires identifying additional GC-specific methylated biomarkers, standardizing pre-analytical conditions, and developing optimized assays, ideally multiplex methylation panels, tailored for early GC detection. Clinically validating these in multicenter trials and making methylation testing cost-effective and accessible will be critical steps for establishing DNA methylation as a useful population-based screening tool to reduce GC mortality.

Lastly, Jin et al. investigated the role of integrin subunit alpha 2 (ITGA2) in pancreatic cancer progression and its effects on the tumor immune microenvironment. Using multiplexed immunofluorescence, the authors found high co-expression of ITGA2, E-cadherin, and PD-L1 in pancreatic tumor tissues. ITGA2 expression positively correlated with pTNM stage, metastasis, and invasion. Meanwhile, ITGA2 inversely correlated with tumor-infiltrating CD4^+^ and CD8^+^ T cells, suggesting an immunosuppressive effect. Knockdown of ITGA2 inhibited pancreatic cancer cell proliferation, migration, and invasion *in vitro*. ITGA2 knockdown also reduced EMT markers and PD-L1 expression. Clinically, high ITGA2 expression is associated with poorer overall survival. Together, these findings identify ITGA2 as a potential driver of pancreatic cancer growth and metastasis, partly by upregulating EMT and immune checkpoint signaling. Targeting ITGA2 may represent a novel therapeutic strategy to suppress tumor progression and enhance anti-tumor immunity in pancreatic cancer. Larger studies are warranted to further evaluate the prognostic and therapeutic value of ITGA2 in this deadly malignancy.

While these findings signify substantial progress, challenges persist in translating genomic biomarkers into widespread clinical adoption. Multi-institutional validation trials, standardization of sample Research Topic and processing protocols, and adherence to ethical and regulatory requirements are essential for successful implementation. Beyond diagnostics and prognosis, integrating genomic analyses with other biomarkers such as proteomics, metabolomics, and imaging can provide comprehensive molecular tumor profiles, facilitating personalized precision medicine. Collaborative efforts and data sharing will be instrumental in accelerating discoveries and implementing genomics-based tests that improve clinical outcomes in real-world cancer care scenarios.

Overall, This Research Topic provides a snapshot of the exciting progress in identifying and applying novel genomic biomarkers to address the diverse and evolving challenges in cancer management.

